# Rewiring of Developing Spinal Nociceptive Circuits by Neonatal Injury and Its Implications for Pediatric Chronic Pain

**DOI:** 10.3390/children3030016

**Published:** 2016-09-20

**Authors:** Mark L. Baccei

**Affiliations:** Pain Research Center, Department of Anesthesiology, University of Cincinnati Medical Center, 231 Albert Sabin Way, Cincinnati, OH 45267, USA; mark.baccei@uc.edu

**Keywords:** pain, neonate, spinal cord, dorsal horn, synapse, glutamate, GABA, glycine, incision, rodent

## Abstract

Significant evidence now suggests that neonatal tissue damage can evoke long-lasting changes in pain sensitivity, but the underlying cellular and molecular mechanisms remain unclear. This review highlights recent advances in our understanding of how injuries during a critical period of early life modulate the functional organization of synaptic networks in the superficial dorsal horn (SDH) of the spinal cord in a manner that favors the excessive amplification of ascending nociceptive signaling to the brain, which likely contributes to the generation and/or maintenance of pediatric chronic pain. These persistent alterations in synaptic function within the SDH may also contribute to the well-documented “priming” of developing pain pathways by neonatal tissue injury.

## 1. Introduction

Pain processing in the central nervous system (CNS) begins in the dorsal horn of the spinal cord which receives direct input from peripheral sensory neurons that are activated by noxious stimuli, defined as stimuli that damage or threaten to damage normal tissue. These signals are integrated with other types of incoming sensory information, including touch, before being transmitted to nociceptive circuits in the brain where the perception of pain ultimately emerges. This need for complex integration of sensory stimuli across multiple modalities is reflected in the functional organization of the dorsal horn network, in that >95% of neurons are propriospinal neurons or local circuit interneurons (both excitatory and inhibitory) whose axons do not leave the spinal cord. Meanwhile, nociceptive information is conveyed to the brain by an exclusive group of projection neurons concentrated mainly in laminae I and V [1], whose firing is strongly controlled by the balance of activity in the different excitatory (glutamatergic) and inhibitory (expressing gamma-aminobutyric acid (GABA) and/or glycine) populations of interneurons. For example, it has long been known that blocking synaptic inhibition within the spinal cord causes robust mechanical allodynia [2,3,4] and unmasks a novel excitatory input to lamina I projection neurons [5,6,7]. Recent evidence suggests that inhibitory interneurons expressing parvalbumin or dynorphin may tonically suppress the activation of ascending projection neurons by innocuous mechanical stimuli, and pharmacogenetic silencing of these interneurons evokes mechanical pain hypersensitivity including allodynia [8,9]. Therefore, a complete mechanistic understanding of pathological pain cannot be obtained without detailed knowledge of how peripheral nerve or tissue damage modifies synaptic transmission within spinal dorsal horn circuits. It is also essential to elucidate the extent to which age determines the effects of injury on synaptic signaling in the spinal superficial dorsal horn. The major aim of this review is to highlight recent work demonstrating that synaptic function within spinal nociceptive networks is persistently influenced by tissue damage during early life.

## 2. Spinal Mechanisms Contributing to Central Sensitization

“Central sensitization” has been operationally defined as an increased responsiveness of nociceptive neurons in the CNS to their normal or subthreshold afferent input, and can drive the generation of chronic pain under pathological conditions [10,11]. It is now clear that a reduction in the efficacy of synaptic inhibition within the spinal dorsal horn is an important contributor to central sensitization after injury. The decreased inhibitory tone in the dorsal horn after nerve or tissue damage reflects a multitude of underlying changes in synaptic function. This includes a dampening of glycinergic transmission [12,13,14] which has been linked to a prostaglandin E2 (PGE2)- and protein kinase A (PKA)-dependent phosphorylation of glycine receptors (GlyRs) containing the α_3_ subunit [15,16]. Following nerve damage, a subset of glycinergic neurons expressing parvalbumin also exhibit a weaker innervation of excitatory protein kinase C gamma (PKCγ)-expressing interneurons in the dorsal horn [8], which likely leads to the disinhibition of this neuronal population previously implicated in neuropathic pain [7,17]. The efficacy of GABAergic and glycinergic inhibition also critically depends on the maintenance of low intracellular Cl^-^ levels within the postsynaptic neuron [18,19], which in turn depends on the activity of the K-Cl co-transporter potassium-chloride transporter member 5 (KCC2) [20,21,22]. Importantly, the expression of KCC2 in adult dorsal horn neurons is significantly reduced by peripheral nerve injury leading to weaker GABAergic inhibition, and in some cases the influence of GABA can switch to being excitatory in nature [23,24]. This injury-evoked shift mimics the situation during early development, where low levels of KCC2 in the dorsal horn [25] lead to a reduced ability to extrude intracellular Cl^-^ [26] and the occurrence of depolarizing responses to GABA [27].

Mounting evidence suggests that while central sensitization occurs at all stages of postnatal development as a consequence of injury, the underlying mechanisms may be at least partially dependent on the age at which the injury occurs [28]. For example, peripheral inflammation or surgical incision of the rodent hindpaw during the first days of life leads to a transient elevation in glutamate release within the superficial dorsal horn (SDH) that is not observed following the same injury at later ages [29]. This enhanced glutamate release includes a strengthening of high-threshold (i.e., putative nociceptive) primary afferent synapses onto lamina II interneurons that requires nerve growth factor (NGF) activation of tropomyosin receptor kinase A (trkA) receptors [30]. The short-term potentiation in glutamatergic function is also activity-dependent, as it is prevented by blocking sciatic nerve activity from the time of injury [31]. In contrast, inflammation during early life failed to compromise synaptic inhibition within the immature dorsal horn [29,31], although Cl^-^ homeostasis was not specifically examined. Peripheral nerve damage also evokes distinct changes in the neonatal versus adult dorsal horn, as no alterations in spontaneous excitatory or inhibitory signaling are seen in the days following sciatic nerve damage at postnatal day (P) 10 [32]. This is interesting in light of behavioral studies showing a delayed onset of neuropathic pain after peripheral nerve injury during early life [33,34].

## 3. Neonatal Injury “Primes” Developing Nociceptive Circuits in the CNS

Tissue damage during a critical period of early life can evoke prolonged changes in nociceptive processing and pain sensitivity. Quantitative sensory testing (QST) approaches have shown that children with a prior stay in a neonatal intensive care unit (NICU) display greater pain sensitivity in response to prolonged noxious stimulation compared to a control, non-hospitalized group, even a decade later [35,36]. These persistent changes were more pronounced in patients that also required neonatal surgery [37]. Nonetheless, the complex nature of the NICU experience makes it difficult to conclusively attribute such long-term changes in pain processing to tissue damage per se, as these infants also experience a high number of stressors [38] that can also modulate nociceptive processing in the CNS. Therefore, it is important to note that preclinical investigations have produced qualitatively similar results. Numerous studies have demonstrated that hindpaw injury during the neonatal period leads to an exacerbated degree of pain hypersensitivity following repeat injury of the affected paw, an effect which persists throughout life [39,40,41,42]. This reflects, at least in part, a localized “priming” of spinal nociceptive circuits following early trauma [43]. Consistent with a role for the spinal cord, neonatal inflammation is sufficient to significantly alter the pattern of gene expression across the adult dorsal horn, including genes that are known to be involved in synaptic transmission [44].

Recent work has explored the long-term effects of neonatal tissue damage on synaptic signaling within the mature SDH. Hindpaw surgical incision at P3 led to a significant dampening of phasic glycinergic transmission onto both GABAergic and presumed glutamatergic interneurons in lamina II of the adult mouse SDH [45]. While this phasic (or “fast”) synaptic inhibition involves the activation of GlyRs located at the synapse [46], the activation of extrasynaptic GlyRs by ambient levels of glycine can evoke a strong tonic inhibition of neuronal excitability in the SDH [47]. Notably, P3 incision also produced a long-lasting decrease in the density of tonic GlyR-mediated current within excitatory lamina II interneurons of the adult spinal cord [45], which is predicted to enhance their firing (via a process of disinhibition). Such deficits in inhibition could be exacerbated if the neonatal injury also persistently reduces KCC2 expression in the dorsal horn, as seen acutely after adult peripheral nerve injury [23,24], although this has yet to be directly investigated. Overall, these results suggest that neonatal tissue injury alters the delicate balance between excitation and inhibition within the mature SDH circuit towards excessive excitation, which in turn would predict an increased level of ascending nociceptive transmission to the brain. However, given that lamina II consists entirely of propriospinal or local circuit interneurons [48,49], the consequences of these changes in synaptic function for pain perception will ultimately depend on the degree to which they influence synaptic signaling onto the spinal projection neurons which convey noxious sensory information to the brain.

## 4. Neonatal Tissue Damage Shapes Synaptic Integration in Adult Spinal Projection Neurons

Primary afferent inputs to the SDH not only directly excite ascending lamina I projection neurons [50,51] but also evoke polysynaptic inhibition of this same population, termed “feedforward” inhibition [52,53], via their synapses onto inhibitory interneurons in the region. Importantly, surgical injury during the neonatal period significantly weakens both GABAergic and glycinergic feedforward inhibition onto adult spinal projection neurons [52]. This cannot be explained by a disruption in the normal innervation of mature projection neurons by local inhibitory interneurons, as early injury failed to alter the number of synaptic boutons expressing known markers of GABAergic and glycinergic presynaptic terminals that were in apposition to adult projection neurons [52]. Instead, the weaker feedforward inhibition could reflect an injury-evoked reduction in the intrinsic membrane excitability of GABAergic interneurons in the mature SDH [54]. Meanwhile, the strength of the direct (i.e., monosynaptic) primary afferent input to adult projection neurons was significantly enhanced by hindpaw incision at P3 [52]. Therefore, early tissue damage significantly alters the balance of synaptic excitation vs. inhibition onto the major output neurons of the spinal nociceptive circuit. This would predict that adult projection neurons would fire more robustly in response to sensory input when preceded by an injury during early life. Consistent with this prediction, adult spino-parabrachial (PB) neurons exhibited a greater number of action potentials in response to primary afferent stimulation in mice with neonatal surgical injury as compared to naïve littermate controls [52]. This demonstrates that neonatal tissue damage persistently increases the signaling ”gain” of the mature SDH network, such that peripheral nociceptive input is amplified to a greater degree within the spinal cord before being transmitted to higher pain centers in the brain. Nonetheless, it should be noted that these prior studies have exclusively focused on the lamina I projection neurons innervating the PB nucleus in the brain. It will be important to also elucidate the long-term effects of early injury on synaptic integration within other populations of spinal projection neurons, such as those targeting the periaqueductal gray (PAG) or thalamus [1], as the electrophysiological properties of projection neurons can vary significantly depending on their target in the brain [55,56,57].

## 5. Long-Term Potentiation at Sensory Synapses onto Spinal Projection Neurons

Primary afferent synapses onto spinal projection neurons can be strengthened by repetitive activation [51,55]. This synaptic long-term potentiation (LTP) represents a major mechanism by which ascending nociceptive transmission to the brain can be amplified within the spinal dorsal horn network (for review see [58]). Numerous lines of evidence point to the functional relevance of spinal LTP for chronic pain. For example, LTP can be evoked by both electrical stimulation of sensory inputs to the dorsal horn as well as peripheral tissue or nerve damage [59,60], and the same electrical stimulation protocols that evoke LTP can produce hyperalgesia in rodents [61,62]. Furthermore, pharmacological agents that prevent the generation of spinal LTP also reduce behavioral pain hypersensitivity after injury [58]. Critically, the administration of LTP induction protocols involving high-frequency stimulation has been shown to increase pain sensitivity in humans [63,64,65]. Therefore, one potential mechanism by which neonatal injury could ”prime” mature nociceptive circuits is by persistently facilitating LTP at sensory synapses onto lamina I projection neurons. This could occur by enhancing the magnitude of LTP at these synapses and/or increasing the likelihood that LTP occurs in response to a given sensory input by modulating the timing rules governing activity-dependent synaptic plasticity within the mature dorsal horn.

It is now abundantly clear that the relative timing of presynaptic versus postsynaptic activity profoundly influences synaptic strength in the CNS, a phenomenon referred to as “spike timing-dependent plasticity” or ”STDP” [66,67]. While the precise temporal rules governing STDP vary across different brain regions, the majority of studies report that presynaptic inputs which precede postsynaptic action potential discharge by a brief interval (10–50 ms) undergo LTP (termed “spike timing-dependent LTP” or ”t-LTP”), while those that follow postsynaptic firing undergo long-term depression (LTD) [68]. This raises the possibility that neonatal tissue damage could facilitate LTP at afferent synapses onto adult projection neurons by: (1) increasing the magnitude of synaptic potentiation produced by highly correlated pre- and postsynaptic firing occurring within the optimum timing window for t-LTP; and/or (2) widening the timing window during which the presynaptic and postsynaptic firing must occur in order to evoke t-LTP.

## 6. Neonatal Injury Relaxes the Timing Rules Governing Spike Timing-Dependent LTP in Adult Spinal Pain Circuits

**Figure 1:**
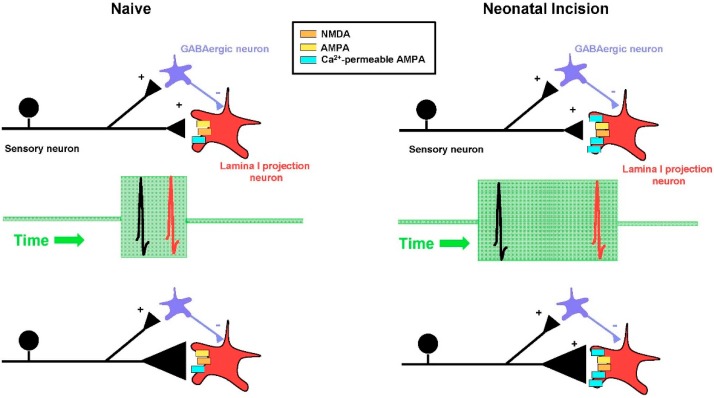
Early tissue damage widens the timing window for evoking spike timing-dependent long-term potentiation (t-LTP) at sensory synapses onto adult spinal projection neurons. **Left column (Naïve)**: In naïve adult mice, highly correlated presynaptic (black) and postsynaptic (red) action potential firing (i.e., occurring within a window of 10–20 ms; green box) led to a strengthening of primary afferent synapses onto ascending lamina I projection neurons. This reflected an increase in the probability of glutamate release from the presynaptic terminals of the sensory neurons, as illustrated here by a larger size of the presynaptic terminals (black triangles in bottom panels). **Right column (Neonatal Incision)**: In adult mice subjected to neonatal surgical injury, the timing window for generating t-LTP (green box) at afferent synapses onto spinal projection neurons significantly widened, such that poorly correlated presynaptic and postsynaptic firing (at pairing intervals ≥50 ms; middle panel) was still able to generate t-LTP. This enhanced propensity to generate t-LTP likely results, at least in part, from an elevated expression of Ca^2+^-permeable α-amino-3-hydroxy-5-methyl-4-isoxazolepropionic acid (AMPA) receptors (AMPARs) (blue rectangles) in mature projection neurons following early tissue damage, since blocking these glutamate receptors prevented t-LTP in neonatally-injured mice but not naïve littermate controls [69]. NMDA: *N*-methyl-D-aspartate.

To address this issue, a recent study [69] evoked action potential firing in lamina I projection neurons at defined intervals either before (i.e., Post → Pre) or after (Pre → Post) the arrival of a presynaptic input mediated by sensory afferents (with the pairing protocol repeated 30 times at 0.2 Hz). The resultant change in synaptic strength was measured and compared between adult mice that had experienced surgical injury as a neonate and naïve littermate controls. In projection neurons from naïve mice, Pre → Post pairings at an interval between 10–20 ms produced significant t-LTP, while the reverse (i.e., Post → Pre) pairings failed to change the amplitude of the synaptic response (i.e., produced neither t-LTP nor t-LTD). Strikingly, hindpaw incision at P3 significantly widened the timing window for evoking t-LTP (Figure 1), as a greater potentiation of excitatory postsynaptic current (EPSC) amplitude was seen in projection neurons from these mice at Pre → Post intervals of 20 and 50 ms compared to the naïve group [69]. In addition, reverse (i.e., Post → Pre) pairings produced marked t-LTP in neonatally-injured mice, suggesting that early tissue damage removes the temporal requirement for the sensory input to precede the firing of adult projection neurons. Such a change is predicted to persistently increase the likelihood that LTP occurs at a given primary afferent synapse, and thus elevate the overall number of synapses that are strengthened following sensory input to the spinal cord. This could favor the excessive amplification of ascending nociceptive transmission to the mature brain in response to subsequent injury and thereby exacerbate chronic pain.

Under normal conditions, the need for sequential Pre → Post activation to produce t-LTP is thought to reflect the biophysical properties of the *N*-methyl-D-aspartate (NMDA) subtype of glutamate receptor (NMDAR), which requires both glutamate binding and membrane depolarization in order to be activated due to a voltage-dependent block of the channel by Mg^2+^ ions at resting membrane potentials [70]. As a result, Pre → Post pairings produce greater Ca^2+^ influx through the NMDAR [71] which is essential for producing t-LTP in many types of CNS neurons [72,73,74]. Interestingly, while the block of NMDARs abolishes t-LTP in adult spinal projection neurons from naïve mice, it fails to do so in neonatally incised mice [69], suggesting a reduced dependence on NMDAR activation in the aftermath of early life injury. Nonetheless, preventing an elevation in intracellular Ca^2+^ within projection neurons prevented t-LTP in both the naïve and P3 incision groups, demonstrating that postsynaptic Ca^2+^ remains a critical mediator of LTP regardless of the presence of noxious sensory experience during the neonatal period. Collectively, these observations raised the possibility that a supplemental source of postsynaptic Ca^2+^ influx is recruited following early tissue injury, thus reducing the reliance on NMDAR activation in order to achieve the intracellular Ca^2+^ levels necessary to drive t-LTP at sensory synapses onto mature projection neurons [69].

Significant glutamate-evoked Ca^2+^ influx into neurons can also occur through a subset of α-amino-3-hydroxy-5-methyl-4-isoxazolepropionic acid (AMPA) receptors (AMPAR) that lack the GluR2 subunit [75,76]. These Ca^2+^-permeable AMPARs are known to be expressed in the superficial dorsal horn and can contribute to the generation of both spinal LTP [77] and chronic pain after injury [78], thereby making them strong candidates to regulate synaptic plasticity within adult spinal nociceptive circuits after early life injury. Indeed, neonatal surgical incision elevated the relative expression of Ca^2+^ permeable AMPARs at sensory synapses onto mature lamina I projection neurons [69]. Furthermore, while blocking Ca^2+^-permeable AMPARs had no effect on timing-dependent LTP (t-LTP) in projection neurons from naïve mice, it completely suppressed t-LTP at the same synapses in neonatally-injured mice [69]. Collectively, the results suggest that early tissue damage unmasks a novel role of Ca^2+^-permeable AMPARs in the regulation of STDP at primary afferent synapses onto ascending projection neurons in the adult spinal cord.

## 7. Future Directions

Despite recent progress towards elucidating the short- and long-term effects of neonatal tissue injury on synaptic signaling within developing spinal nociceptive circuits, many important questions remain unanswered. For example, how does early tissue damage alter the synaptic “microcircuits” within the mature SDH? While prior work has demonstrated reduced feedforward synaptic inhibition onto adult projection neurons after neonatal surgical injury, how this injury influences neurotransmitter release from specific subpopulations of inhibitory dorsal horn interneurons (i.e., those expressing parvalbumin, neuropeptide Y, galanin or nitric oxide synthase) remains unknown. In addition, since immune cells play an important role in shaping synaptic development in the CNS [79] and early tissue damage alters neuroimmune signaling in the mature spinal cord [43], it will be interesting to examine the potential role of spinal microglia in orchestrating the short- and long-term alterations in synaptic function within the SDH after neonatal injury. Microglia could also contribute to the changes in spike timing-dependent plasticity within adult spinal projection neurons after early tissue damage, as they are known to modulate other forms of LTP in the SDH [80].

The t-LTP characterized at primary afferent synapses onto ascending projection neurons appears to involve the release of a retrograde messenger which enhances glutamate release from the sensory neurons [69]. However, the identity of the retrograde signal, and whether neonatal injury alters this signaling pathway, has yet to be investigated. Another intriguing question is whether early tissue damage evokes a novel timing window at afferent synapses onto adult projection neurons, or whether this more permissive environment for t-LTP normally exists during early life and the injury somehow prevents a developmental sharpening (or “tuning”) of the timing window. Notably, the prolonged changes in pain sensitivity [41,42] and synaptic plasticity [69] both require that the initial injury occur during a critical period of early postnatal development, corresponding to the first postnatal week in the rodent. However, the mechanisms which underlie the closure of this critical period are currently a mystery. A better understanding of why tissue damage at later ages fails to evoke the same permanent alterations in spinal nociceptive processing could yield valuable insight into novel strategies to minimize the persistent effects of neonatal injuries on developing nociceptive pathways.
